# HighRPD: A high-altitude drone dataset of road pavement distress

**DOI:** 10.1016/j.dib.2025.111377

**Published:** 2025-02-07

**Authors:** Jin He, Liting Gong, Chuan Xu, Pin Wang, Yiyong Zhang, Ou Zheng, Guanghe Su, Yufeng Yang, Jialin Hu, Yuchen Sun

**Affiliations:** a Shanxi Provincial Innovation Center of Digital Road Design Technology, Taiyuan 030000, PR China; b National United Engineering Laboratory of Integrated and Intelligent Transportation, National Engineering Laboratory of Integrated Transportation Big Data Application Technology, School of Transportation and Logistics, Southwest Jiaotong University, Chengdu 611756, PR China; c InnoViX Lab, Sichuan Zhiling Technology Co., Ltd. Chengdu 610040, PR China

**Keywords:** Unmanned aerial vehicle (UAV), Pavement distress, Crack, Pothole, Object detection, Computer vision

## Abstract

This dataset presents pavement distress data collected using high-altitude Unmanned Aerial Vehicles (UAVs) over road networks in Shanxi, China. The data collection involved capturing aerial images of road pavements with UAVs flying at high altitudes to efficiently cover large areas. A total of 11,696 high-resolution road pavement images were acquired and annotated with detailed distress information: 12,365 line annotations indicating linear cracks, 8239 block annotations marking block cracks, and 1412 pit annotations identifying potholes. Named HighRPD, this extensive dataset addresses the scarcity of publicly available UAV-based road pavement distress datasets, which are currently limited in data volume. HighRPD offers a substantial number of samples compared to existing public datasets, providing a valuable resource for developing and benchmarking pavement distress detection algorithms. Additionally, the dataset offers data scientists and machine learning engineers a rich repository of road surface data, facilitating the development and training of models for image recognition, pavement condition classification, and object detection. Consequently, HighRPD supports applied research in areas such as transportation and urban planning.

Specifications Table

**Table:** 

Subject	Computer Vision and Pattern Recognition, Computer Science Applications
Specific subject area	This dataset focuses on pavement disease, specifically line, potholes, and block, aiming to improve the accuracy of pavement disease detection and analysis, and to provide strong data support for road maintenance and management*.*
Type of data	2D-RGB Images(.jpg), Labels in YOLO Format(.txt), Raw
Data collection	Equipment Used: Dataset captured using a DJI M300 drone with a Zenmuse P1 camera (35 mm lens). Data Collection Details: The drone operated at an altitude of approximately 50 m above ground. Original road pavement images were captured at a resolution of 8192×5460 pixels. Image Processing: A total of 546 large original images were screened. Images were uniformly cropped into smaller 640×640 pixel tiles. Black padding were added to fit the target size. Annotation and Verification: The Labelbox tool was used to create TXT files for annotating pavement distress in the images. After multiple rounds of annotation and verification, a total of 11,696 road pavement images were successfully labeled.''.
Data source location	*Shanxi Province, China* *The GPS coordinates: The beginning and ending of the road segment are (37.768°*N*, 112.772°E), (37.771°*N*, 112.762°E).*
Data accessibility	Repository name: Mendeley Data Data identification number: 10.17632/sywswj7djj.1 Direct URL to data: https://data.mendeley.com/datasets/sywswj7djj/1 Instructions for accessing these data: Shanxi Provincial Innovation Center of Digital Road Design Technology, Southwest Jiaotong University
Related research article	

## 1 Value of the Data

• **Significance of UAV-Based Detection**: With the increasing road maintenance demands, cost-effective methods for detecting pavement distress are critical. UAVs, combined with deep learning, offer advantages such as low cost, easy deployment, large-area coverage, and enhanced safety by avoiding risks to inspection personnel. However, the lack of drone-based pavement distress datasets has limited research in this area. This dataset addresses this gap and supports advancements in UAV-based pavement distress detection.
• **Unique Dataset Characteristics**: Existing open-source datasets for pavement distress detection are limited in size and diversity. The HighRPD dataset, consisting of standardized 640×640 pixel images, is a relatively large and diverse UAV-based dataset. It significantly expands the volume and variety of data for pavement distress detection.
• **Utility for Data Science and ML Applications**: The dataset offers a rich resource for Data Scientists and Machine Learning Engineers, enabling the development and training of models for image recognition, pavement condition classification, and object detection. These models can drive road maintenance.

## 2 Background

Road networks face increasing distress due to traffic, affecting safety and serviceability. Distress types include line (cracks), block (dense cracks), pit (potholes), etc. Traditional detection methods are inefficient and subjective. Modern methods using sensor-equipped vehicles are accurate but costly. UAVs and deep learning offer a cost-effective, safer alternative. Among the recent studies, Zhang et al. [1] enhanced the YOLO v3 model with an attention mechanism to detect cracks, repairs, and potholes in 2401 UAV images of road pavement distress (500×500 px²). Hassan et al. [2] added three convolutional layers to YOLO v3Tiny and applied it to detect road pavement distress in 240 UAV images (416×416 px²). Zhu et al. [3] compared Faster R-CNN, YOLO v3, YOLO v4, and variations with different backbones for detecting road pavement distress in UAV images. In their experiment, they used a total of 3151 images (512×512 px²) that included transverse cracks, longitudinal cracks, diagonal cracks, alligator cracking, potholes, and repairs. Silva et al. [4] compared the performance of YOLO v5, YOLO v7, and YOLO v5 combined with transformers for this task. Silva's approach, which incorporated random rotation as a data augmentation technique, used a total of 4873 images (640×640 px²) covering longitudinal cracks, alligator cracking, potholes, bulging, and repairs. Wu et al. [5] applied the Faster R-CNN model to detect longitudinal cracks in both normal and infrared thermal images of road pavement distress, using a total of 2088 images. However, data scarcity limits model development. Road pavement distress Prominent datasets such as crack500 [6,7], GAPs384 [7,8], cracktree200 [9], CFD [10], AsphaltCrack300 [11], AEL [12], CrackLS315 [13], among others, are primarily labeled for line crack detection. However, these datasets exhibit ambiguity in categorizing some line pavement distress that fall into the block category. Additionally, in the case of road pavement distress datasets annotated with bounding boxes from a drone perspective, such as the Global Road Damage Detection road pavement distress dataset (RDD) [14], only 1919 cases of road pavement distress were selected after screening and inspection. Similarly, the UAPD road pavement distress dataset [15] contained just 1882 cases of road pavement distress. The UAV-PDD2023 [16] dataset are obtained through drone-based aerial imaging of pavement surfaces, contains 11,158 instances and is relatively large compared to similar datasets, but for detecting pavement distress using deep learning framework, larger-scale datasets can improve model training performance more effectively. The lack of datasets is a major barrier for UAV-based pavement distress detection. To enrich the datasets, this paper introduces a larger dataset to facilitate research and development in this area.

## 3 Data Description

The HighRPD image dataset consists of 11,696 images with a resolution of 640×640 pixels., annotated with three main types of pavement distress: line cracks, block cracks, and potholes. The annotations are distributed as follows: 12,365 line crack annotations, 8239 block crack annotations, and 1412 pothole annotations.

**Fig. 1:**
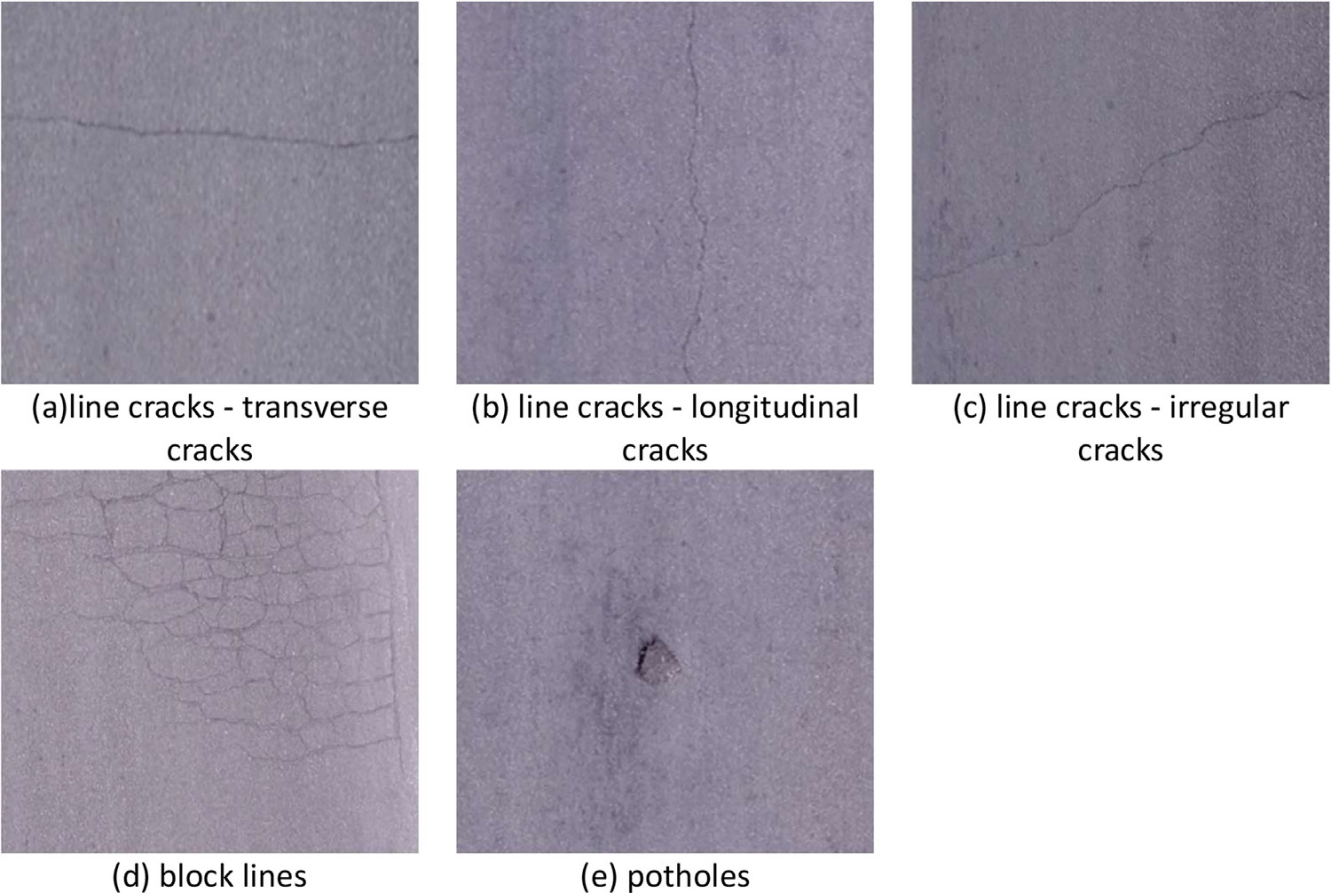
Sample images from the dataset.

**Fig. 2:**
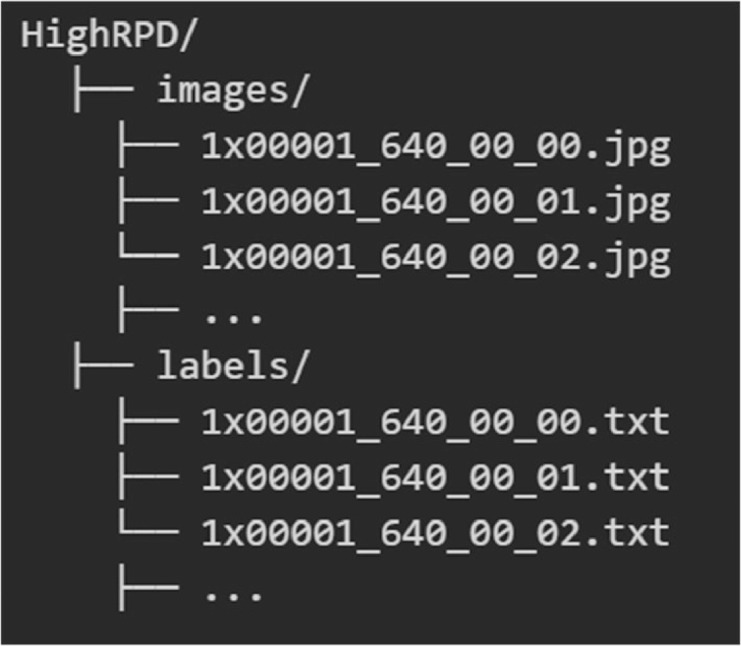
The directory structure of the dataset.

Line cracks are categorized into line cracks - transverse cracks, line cracks - longitudinal cracks, and line cracks - irregular cracks. Line cracks - irregular cracks are classified as cracks that are neither transverse nor longitudinal, including shapes such as U-shaped, W-shaped, and H-shaped cracks. Block cracks are pavement cracks that form rectangular block-like regions, typically resulting from the intersection of transverse and longitudinal cracks. Potholes are depressions formed on the pavement surface due to localized aggregate loss. Sample images are shown in Fig. 1, and the directory structure of the dataset is illustrated in Fig. 2.

The dataset is organized into two main subfolders: ‘images’ and ‘labels’. The ‘images’ folder contains the 640×640 pixel JPG images, while the ‘labels’ folder contains corresponding text files, formatted according to the YOLO annotation style. Each object in the images is described in a single line within these label files, e.g., (0 0.2953125 0.5 0.084375 1.0). The first value “0” indicates that the object class is “line crack”, while “1” and “2” represent “block cracks” and “potholes”, respectively. The second value “0.2953125” represents the x-coordinate of the center of the bounding box, normalized by the image width (i.e., x_center / image_width). The third value “0.5” represents the y-coordinate of the bounding box center, normalized by the image height (i.e., y_center / image_height). The fourth value “0.084375” indicates the width of the bounding box, normalized by the image width (i.e., width / image_width). The fifth value “1.0” represents the height of the bounding box, normalized by the image height (i.e., height / image_height). These coordinates are normalized by dividing the x and width by the image width, and the y and height by the image height.

## 4 Experimental Design, Materials and Methods

**Fig. 3:**
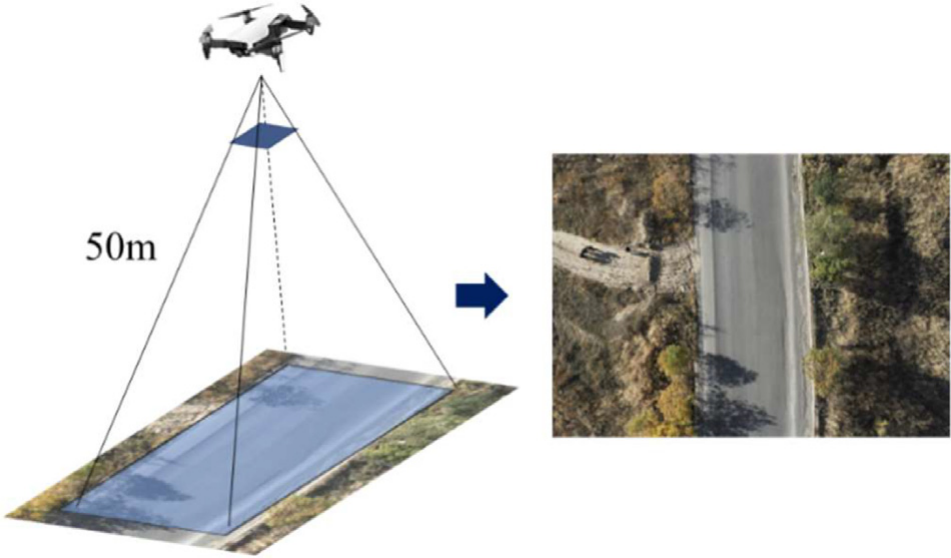
Illustrates the drone capturing images of the road.

The images were captured using the DJI M300 RTK drone equipped with a 35 mm lens from the DJI Zenmuse P1 camera, with a resolution of 45 megapixels and an image size of 8192×5460 pixels. To ensure effective detection of smaller pavement distresses, the flight height was tested at various altitudes. Considering the potential safety risks of flying too low, the final flight height was 50 m. All images were captured during the daytime, with clear weather, ensuring no rainfall in the previous three days to avoid any standing water on the road surface (Fig. 3).

**Fig. 4:**
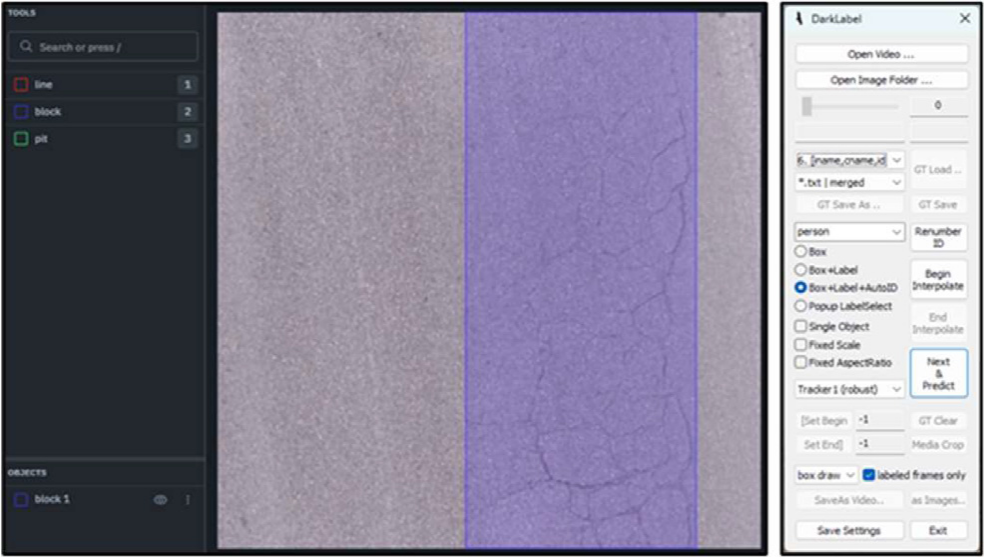
Dataset building tools (Left: Labelbox, Right: Darklabel).

**Fig. 5:**
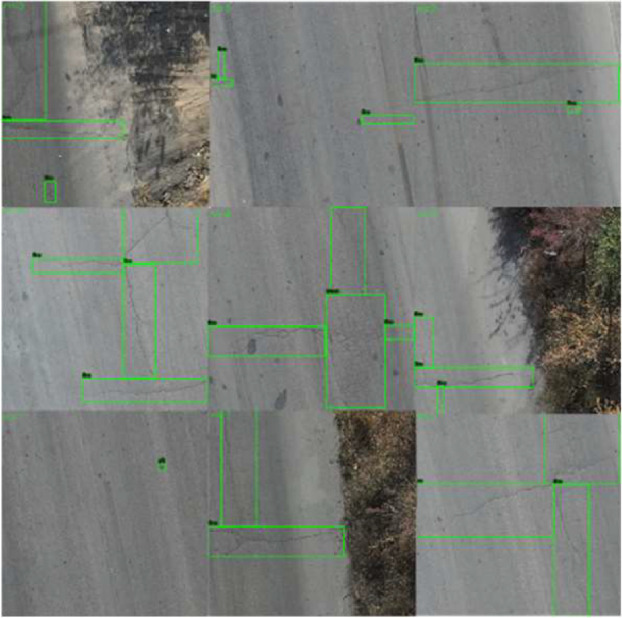
HighRPD dataset sample.

**Fig. 6:**
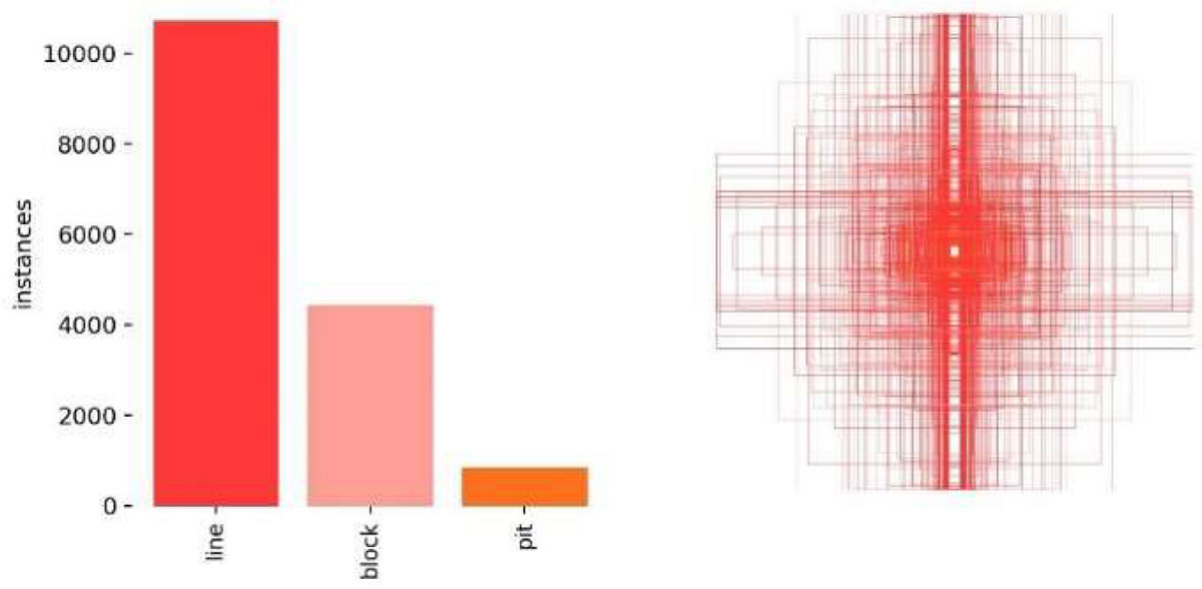
Specifics of labeling self-built datasets.

Since the original images were large, they were cropped to a uniform size of 640×640 pixels, with black padding added where necessary to fit the size. The uniform size of 640×640 was chosen because the object detection model YOLO v8 we used supports input data of this fixed size. After filtering out images with no pavement distress, a total of 11,696 valid images were obtained. The dataset was constructed using the Labelbox platform [17] combined with DarkLabel tool [18] (see Fig. 4). Fig. 5 shows some examples of annotation results, while Fig. 6 illustrates the overall appearance frequency distribution. The left panel displays the sample size for each annotation category, and the right panel shows the distribution of the basic shapes of the bounding boxes. Overall, cracks are the most common type of pavement distress in the annotated data, and they are mostly represented by narrow rectangular bounding boxes.

As the HighRPD dataset expands the sample size of pavement distresses, our dataset demonstrates significant improvements in the identification accuracy of various types of road distresses compared to existing open-source datasets. For datasets like UAPD [19] and RDD China Drone [14], which distinguish cracks based on their orientation, we have consolidated these cracks into a single category (crack). Table 1 provides detailed statistics of the characteristics of each dataset.

**Table 1:** Summary of different datasets in road pavement distress.

Dataset	Image Number	Image Size	Annotation number		
			line	Block	pit
Crack500 [20]	676	640×360	676	0	0
GAPs384 [21]	509	540×640 540×440	502	7	0
Cracktree200 [22]	206	800×600	206	0	0
CFD [23]	566	480×320	526	40	0
AsphaltCrack300 [11]	795	504×672	795	0	0
CrackLS315 [24]	315	512	311	4	0
UAPD [19]	1882	512×512	2520	319	67
**HighRPD**	**11,266**	**640×640**	**12,611**	**8158**	**1187**

## Limitations

• The detection performance of the pit category in road pavement distress should be improved. Although the HighRPD dataset we developed shows a significant increase in the number of labels for the pit category compared to previous studies, it still represents less than 5 % of the total data we annotated. Regarding the detection model's actual performance, this distress category demonstrates significantly lower effectiveness. Therefore, optimization for the pit category is essential, which could involve increasing the volume of labeled data or improving and integrating multiple object detection models specifically tailored for small targets, such as the pit category of road pavement distress.
• Incorporate a module for extracting pixel-level information on road pavement distress road pavement distress. In practical applications, obtaining pixel-level details of road pavement distress is crucial, especially for tasks such as evaluating the detailed severity of road pavement conditions. Hence, we plan to integrate edge detection methods and segmentation models into our future work on road pavement distress.

## Ethics Statement

The authors hereby declare that we have thoroughly reviewed the ethical guidelines for submitting research to Data in Brief. We affirm that this submission does not pertain to human or animal testing, nor does it include any data sourced from social media platforms. Our work strictly conforms to the ethical frameworks outlined by the journal.

## CRediT Author Statement

**Jin He:** Conceptualization, Methodology, Supervision. **Liting Gong:** Data curation, Writing, Original draft preparation. **Chuan Xu:** Conceptualization, Methodology, Supervision. **Pin Wang:** Data curation, Writing, Original draft preparation. **Yiyong Zhang:** Data collection, Investigation. **Ou Zheng:** Conceptualization, Methodology, Supervision. **Guanghe Su:** Data collection, Investigation. **Yufeng Yang:** Data curation, Visualization. **Jialin Hu:** Data curation, Visualization. **Yuchen Sun:** Data collection, Investigation.

## Data Availability

Mendeley DataHighRPD (Original data).
